# VariantDetective: an accurate all-in-one pipeline for detecting consensus bacterial SNPs and SVs

**DOI:** 10.1093/bioinformatics/btae066

**Published:** 2024-02-15

**Authors:** Philippe Charron, Mingsong Kang

**Affiliations:** Ottawa Laboratory-Fallowfield, Canadian Food Inspection Agency, 3851 Fallowfield Road, Nepean, Ontario K2J 4S1, Canada; Ottawa Laboratory-Fallowfield, Canadian Food Inspection Agency, 3851 Fallowfield Road, Nepean, Ontario K2J 4S1, Canada

## Abstract

**Motivation:**

Genomic variations comprise a spectrum of alterations, ranging from single nucleotide polymorphisms (SNPs) to large-scale structural variants (SVs), which play crucial roles in bacterial evolution and species diversification. Accurately identifying SNPs and SVs is beneficial for subsequent evolutionary and epidemiological studies. This study presents VariantDetective (VD), a novel, user-friendly, and all-in-one pipeline combining SNP and SV calling to generate consensus genomic variants using multiple tools.

**Results:**

The VD pipeline accepts various file types as input to initiate SNP and/or SV calling, and benchmarking results demonstrate VD's robustness and high accuracy across multiple tested datasets when compared to existing variant calling approaches.

**Availability and implementation:**

The source code, test data, and relevant information for VD are freely accessible at https://github.com/OLF-Bioinformatics/VariantDetective under the MIT License.

## 1 Introduction

Variant calling is a process used in genomics to identify and compare genetic differences between multiple individuals or samples (Olson *et al.* 2023). While single nucleotide polymorphisms (SNPs) and small insertions and deletions (INDELs) have been extensively studied for disease outbreak monitoring and epidemiological studies (Schork *et al.* 2000, Paranthaman *et al.* 2021), the investigation of structural variants (SVs), such as inversions, duplications, translocations, and large insertions or deletions, has been relatively limited, especially in prokaryotes. These SVs have a more significant impact on adaptation and evolution (West *et al.* 2022). Additionally, due to the high diversity of bacterial genomes, even within the same species, the performance of various variant callers can vary significantly (Bush *et al.* 2020).

To ensure the utmost accuracy in variant calling, it is common practice to obtain agreement results from multiple variant calling tools for both SNPs (Semegni *et al.* 2011, Chiara *et al.* 2018, Koboldt 2020) and SVs (Mohiyuddin *et al.* 2015, Becker *et al.* 2018, Chilinski and Plewczynski 2022). However, there are currently no dedicated consensus variant callers available specifically for identifying bacterial SNPs and SVs simultaneously using raw read files (FASTQ) or genome assembly data files (FASTA), which limits the use of publicly available assembly data. To address this issue, we developed VariantDetective (VD), an all-in-one bioinformatics pipeline that detects both SNPs and SVs using various file formats as input.

## 2 Methods

### 2.1 Design and implementation

The VD pipeline, implemented in Python, aims to generate consensus variant calls from three different tools for SNPs/INDELs: Freebayes v1.3.5 (Garrison and Marth 2012), GATK HaplotypeCaller v4.2.6.1 (McKenna *et al.* 2010), and Clair3 v.0.1-r11 (Zheng *et al.* 2022). Similarly, it incorporates four different pipelines for SVs: NanoVar v1.3.9 (Tham *et al.* 2020), NanoSV v.1.2.4 (Cretu Stancu *et al.* 2017), CuteSV v1.0.13 (Jiang *et al.* 2020), and SVIM v.1.4.2 (Heller and Vingron 2019). The pipeline consists of multiple steps, including data preparation, variant calling, and the identification of consensus variants with the detailed procedures and rationales explained in Supplementary material.

In the current version, VD accepts short-read sequencing raw data for identifying SNPs/INDELs (FASTQ), long-read sequencing raw data (FASTQ) for SV analysis, and genome assemblies in FASTA format for simultaneous predictions of SVs, SNPs, and INDELs. Additionally, VD offers the “combineVariants” module (Supplementary Fig. S1) to use VCF files produced by different variant callers, versions, or parameters as input for users’ specific requirements. This module seamlessly integrates these external VCF files, ensuring that users can benefit from the consensus variant calling approach of VD while maintaining the flexibility to employ customized variant detection strategies. The VD pipeline offers outputs in multiple formats, including VCF, CSV, and TSV files, as well as a summary file outlining the amount of different variant types found within a sample. Furthermore, the results generated by each individual caller are saved in dedicated output folders.

### 2.2 Resources

The whole genome sequence from *Burkholderia mallei* (*B. mallei*) ATCC 23344 (GCF_000011705.1), which contains a highly plastic genome (Nierman *et al.* 2004), was used to generate five different simulated datasets. SURVIVOR v1.0.7 (Jeffares *et al.* 2017) was used to simulate SNPs at a rate of 0.00017 variants per base pair, as well as 100 SVs. These numbers were determined based on variant rates observed in *B. mallei* completed and chromosome-level genome assemblies available in the RefSeq database (accessed on 2023–05-01), which contain both short-read and long-read raw data (Supplementary Table S1). Previous studies also demonstrated that genomic GC content affects the performance of SNP/INDEL calling (Zhao *et al.* 2020, Barbitoff *et al.* 2022). In this study, five additional simulated datasets were generated using not only the high GC content (68.5%) *B. mallei* genome, but also the low GC content bacteria *Lactobacillus acidophilus* (*L. acidophilus*) str. La-14 (CP005926.2) with a GC content of 34.5% and the moderate GC content bacteria *Escherichia coli* (*E. coli*) str. K-12 substr. MG1655 (NC_000913.3) with a GC content of 50.5%. In total, 15 simulated datasets were created.

Additionally, widely used benchmarking SNPs and SVs datasets were acquired from https://ora.ox.ac.uk/objects/uuid:8f902497-955e-4b84-9b85-693ee0e4433e (Bush *et al.* 2020) and http://ftp.1000genomes.ebi.ac.uk/vol1/ftp/technical/working/20131209_na12878_pacbio/si/ (Genomes Project *et al.* 2015, Parikh *et al.* 2016) respectively.

## 3 Results and discussion

Our VD pipeline has multi-threading implemented, which reduces the runtime if resources are available (Supplementary Fig. S2). Additionally, it has the ability to handle large amounts of genomic variant data and high sequencing coverage (Supplementary Figs S3 and S4).

To evaluate the optimization and performance of the consensus variant set, we used five independent simulated datasets for each bacterial genome that was evaluated. Regardless of the species that was investigated, the agreement from at least two callers for SNP/INDEL identification was required to achieve a high level of accuracy with F1 scores over 0.99 in all simulated datasets (Supplementary Fig. S5A, C, E, and G). This result aligns with a previous study that employed two callers to generate benchmarking data for small genomic variants (Bush *et al.* 2020). Similarly, our results for SV detection showed superior levels of accuracy when two or three callers were used, compared to performances of VD using one or four callers (Supplementary Fig. S5B, D, F, and H). Consequentially, the VD pipeline employs a default setting of two callers for SNP/INDEL identification and three callers for SV identification.

**Figure 1.**
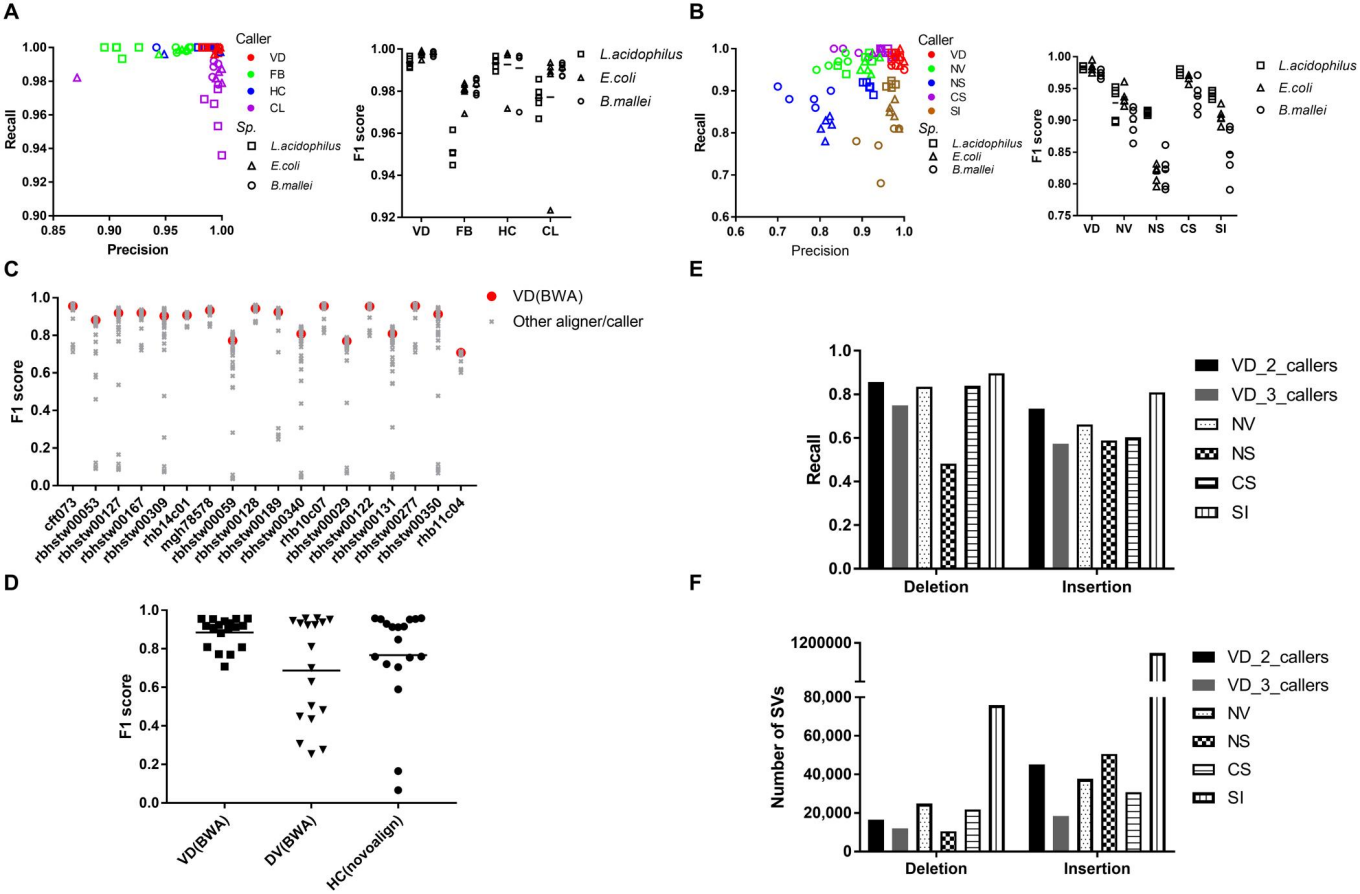
Performance and benchmarking of VD. (A, B) Benchmarking of VD against three SNP callers (A) and four SV callers (B) using 15 simulated datasets. SURVIVOR was used to simulate SNPs and different types of SVs in the assembly of representative *L. acidophilus*, *E. coli*, and *B. mallei* genomes. (C, D) Comparison of F1 scores of VD with 41 SNP calling pipelines (C). Overall accuracy of VD against two top SNP callers: DV and HC (D). F1 scores of 41 SNP calling pipelines, including DV and HC, were obtained from a previous study (Bush *et al.* 2020). (E, F) Performance of VD against four SV callers using benchmarking human SVs datasets, including recall (E) and the number of predicted SVs (F). Parameters used for each caller were taken from a previous benchmarking publication (Tham *et al.* 2020). The 2 or 3 callers represent the results obtained from a minimum consensus of two or three callers used to generate the final variant list. VD: VariantDetective; FB: Freebayes; HC: GATK HaplotypeCaller; CL: Clair3; NV: NanoVar; NS: NanoSV; CS: CuteSV; SI: SVIM; DV: DeepVariant.

VD’s performance was evaluated and benchmarked against three SNP/INDEL callers (Freebayes, HaplotypeCaller, and Clair3), and four SV callers (NanoVar, NanoSV, CuteSV, and SVIM) by assessing their accuracy in predicting SNPs and SVs on 15 simulated genomic variant datasets. VD proved to be more accurate than other tools, achieving the highest average F1 scores of 0.996 for SNP, followed by HaplotypeCaller with a mean F1 score of 0.992 using reads mapped to the original genome from which the mutated reads were generated (Fig. 1A). The impact of mapping simulated reads to the original genome, where in silico mutations were introduced, and to a “divergent” reference genome was also tested. When employing the divergent genome as a reference, compared to the simulated genome as a reference, a decrease in the mean F1 score of VD from 0.999 to 0.942 was observed (Supplementary Fig. S6A and B). This decline in performance is consistent with prior research (Bush *et al.* 2020). Nevertheless, despite the decrease in F1 score, VD continued to outperform other tools, as demonstrated in Supplementary Fig. S6.

Similarly, VD also showed highly accurate and robust SV identifications with the highest average F1 score of 0.974, followed by CuteSV with a mean F1 score of 0.955 (Fig. 1B). While all other SV callers were able to identify the different types of SVs, they had higher rates of false positives compared to VD in the *B. mallei* simulated datasets, especially with inversions, deletions and insertions (Supplementary Table S2).

Finally, the same set of tools was tested on 18 different real and validated SNP datasets used in a previous study (Bush *et al.* 2020). This dataset is comprised of different bacterial species, namely *E. coli*, *Klebsiella pneumonia*, *Klebsiella oxytoca*, *Enterobacter cloacae*, *Enterobacter kobei*, and *Citrobacter freundii*. We ran VD using both the assembled genomes (FASTA) and raw sequencing data (FASTQ) as input to track the effects of input type on variant calling. Except for Freebayes, all other callers (VD, HaplotypeCaller, and Clair3) performed similarly when using FASTQ inputs, exhibiting average F1 scores above 0.85 (Supplementary Fig. S7A). In comparison, VD and HaplotypeCaller both performed similarly when using FASTA inputs, with Freebayes and Clair3 achieving lower F1 scores (Supplementary Fig. S7B). VD exhibits a slightly better performance over HaplotypeCaller in both analyses (Supplementary Fig. S7A and B). Specifically, VD shows a higher F1 score compared to HaplotypeCaller in all the datasets when using FASTQ inputs (Supplementary Fig. S7A). Additionally, when comparing the performance of VD using two different aligners, it was observed that VD using the BWA aligner performed slightly better on short-read sequencing data for several real datasets (Supplementary Fig. S8). This suggests that the BWA aligner outperformed minimap2 in handling complex short-read raw data generated from the Illumina platform. This observation aligns with previous findings (Barbitoff *et al.* 2022), although no significant difference was previously noted between these two aligners. When compared to F1 scores of 41 SNP calling pipelines that are combinations of five aligners (BWA, minimap2, Novoalign, Snippy, and Stampy) and 11 variant callers (16GT, Freebayes, HaplotypeCaller, LoFreq, mpileup, Platypus, SNVer, SNVSniffer, Strelka, VarScan, and Snippy) using the same benchmarking SNP datasets (Bush *et al.* 2020), VD consistently demonstrated high and stable F1 scores across all datasets ranging from 0.71 to 0.96, with an average of 0.89 (Fig. 1C). Notably, VD outperformed DeepVariant and HaplotypeCaller (Fig. 1D), which were shown to have the best performance and highest robustness in previous studies (Bush *et al.* 2020, Barbitoff *et al.* 2022). Additionally, by comparing the accuracy of VD using short-read sequencing raw reads (FASTQ files) with genome assembly data files (FASTA files) as input using the 18 real bacterial datasets, no considerable difference was observed (Supplementary Fig. S9).

Despite the lack of any validated benchmarking datasets specifically designed for SVs in bacteria, we assessed the sensitivity of VD and four individual callers employed within VD using a benchmarking dataset created for human SVs (Parikh *et al.* 2016). SVIM exhibited the highest recall for deletions and insertions, followed by VD with two consensus callers and NanoVar. However, SVIM detected over 3 times and 20 times more deletions and insertions, respectively, which could potentially compromise its specificity. It is worth noting that not all SVs within this benchmark dataset have been discovered (Tham *et al.* 2020), making it challenging to fully evaluate the precision and F1 scores of these tools. Additionally, due to the complexity of SVs and the limited availability of validated real benchmarking datasets, it is very difficult to perform a comprehensive benchmarking study for SV callers with multiple real datasets. This is definitely an area of improvement for future work, particularly for bacterial datasets.

In conclusion, VD is a dependable and comprehensive bioinformatics pipeline, which is proficient at making genomic variant calls and managing diverse input file types. Its versatile features have the potential to be advantageous for bacterial genomics, epidemiological studies, and outbreak investigations in the forthcoming years.

## Supplementary Material

btae066_Supplementary_Data
