# Supplementary material for: VariantDetective: an accurate all-in-one pipeline for detecting consensus bacterial SNPs and SVs
Source: Bioinformatics. 2024 Feb 15;40(2):btae066. doi: 10.1093/bioinformatics/btae066 (PMC10898327; doi:10.1093/bioinformatics/btae066)
Supplement: btae066_Supplementary_Data [file btae066_supplementary_data.docx]

**Supplementary information for:**

**VariantDetective: an accurate all-in-one pipeline for detecting consensus bacterial SNPs and SVs**

Philippe Charron* and Mingsong Kang*

Ottawa Laboratory-Fallowfield, Canadian Food Inspection Agency, Ottawa, ON, Canada

*To whom correspondence should be addressed:

Philippe Charron : [phil.charron@inspection.gc.ca](mailto:phil.charron@inspection.gc.ca)

Mingsong Kang : [mingsong.kang@inspection.gc.ca](mailto:mingsong.kang@inspection.gc.ca)

**Supplementary Methods**

**1. Detailed description of VariantDetective algorithm**

- 1. **Input**

VariantDetective (VD) accepts various types of files as input (Figure S1) and requires a reference genome assembly (FASTA file) to initiate an analysis. In its current version, if an assembled genome (FASTA file) is provided as query input, VD will generate simulated long-read data using modified code from Badread v0.2.0 (<https://github.com/rrwick/Badread>) with default settings set to an average fragment length of 15000 bp ± 13000 bp and 50X read depth. Then the simulated long-read FASTQ file is used to detect different types of variants, including SNPs, INDELs, and SVs. If long-read data (FASTQ) is used as input, VD will only initiate the SV calling. In contrast, if paired-end short reads data (FASTQ) generated from Illumina platforms are provided, VD will only predict short variants (SNPs and INDELs). Moreover, VCF files are also accepted as input to initiate the “combineVariants” module (Figure S1). It is worth noting that all input files can be supplied in the gzipped format as well.

- 1. **Alignment**

VD incorporates two different aligners, either BWA v0.7.17 (Li and Durbin, 2009) or minimap2 v2.24 (Li, 2016) in the current version to map paired-end short reads to the reference genome. The default aligner for SNP/INDEL calling is BWA. Regardless of the chosen aligner, SAMtools v1.10 (Li, et al., 2009) is used to process and sort reads. However, if a genome assembly is used as input, the simulated long reads are exclusively mapped using minimap2. After the mapping step, additional processing steps generate the required files for further analyses using Picard v2.27.3 (<https://broadinstitute.github.io/picard/>) and SAMtools v1.10.

- 1. **SNP and INDEL calling**

A recent study aimed to benchmark various SNP/INDEL callers, specifically using bacterial genome sequencing data, found that Freebayes and GATK HaplotypeCaller consistently delivered the highest performance (Bush, et al., 2020). Furthermore, two deep learning-based callers, Clair3 and DeepVariant, were also found to be highly accurate and robust, with the ability to work with both short- and long-read data, but Clair3 was particularly noted for its faster processing time (Barbitoff, et al., 2022; Zheng, et al., 2022). Therefore, the SNP/INDEL calling module of VD is performed using Freebayes v1.3.5 (Garrison and Marth, 2012), GATK HaplotypeCaller v4.2.6.1 (McKenna, et al., 2010), and Clair3 v0.1-r11 (Zheng, et al., 2022). Once variant calling is complete, low-quality variants are filtered through the "--minqual_snp" parameter achieved using vcffilter v1.0.2, with the default minimum quality threshold being set to 20 (Molina-Mora and Solano-Vargas, 2021). In addition, VD offers the "combineVariants" module to use VCF files produced by different SNP/INDEL callers, versions, or parameters as input. This module enhances the system's flexibility and allows users to tailor the process to their particular needs.

To create a consensus set of variants, all VCF files are compressed using bgzip v1.10.2 (<http://www.htslib.org/doc/bgzip.html>) and indexed using tabix v1.10.2 (http://www.htslib.org/doc/tabix.html). VCF file intersections are generated using vcftools v0.1.16 (<https://vcftools.github.io/index.html>) and concatenated using bcftools v1.13 (<https://samtools.github.io/bcftools/>). The final VCF consensus file contains variants found in at least two variant callers when using default settings.

- 1. **SV calling**

The discovery and accurate characterization of SVs rely heavily on long-read sequencing and SV callers (Jiang, et al., 2021). The Oxford Nanopore Technologies sequencing platform, in particular, is cost-effective, fast, and portable, making it an attractive choice for SV investigation (Wang, et al., 2021). Various popular SV callers have been widely used for Nanopore sequencing data, including NanoSV, Sniffles, SVIM, cuteSV, and NanoVar (Romagnoli, et al., 2023). Due to the complexity of SV identification, the performance of these tools may vary based on the characteristics of the data and specific types of SV you are interested in. It's advisable to run different tools on your data and compare their results to minimize caller-related bias (Yang, 2020). While NanoSV (Cretu Stancu, et al., 2017) and NanoVar (Tham, et al., 2020) are designed for Nanopore sequencing data, they can also work well with other long-read technologies. Moreover, multiple studies have shown that SVIM and cuteSV outperform Sniffles, particularly in terms of enhanced sensitivity for datasets with low coverage (Bolognini and Magi, 2021; Heller and Vingron, 2019; Jiang, et al., 2020; Liu, et al., 2022). Although Sniffles has an updated version called Sniffles2, published earlier this year (Smolka, et al., 2024), further independent evaluations are still needed to assess its performance. Therefore, for the effective identification of SVs using VD, the SV calling process involves using NanoVar v1.3.9 (Tham, et al., 2020) for both real or simulated reads. NanoVar uses minimap2 for read mapping and performs processing steps. The mapped reads obtained from NanoVar serve as input for each subsequent variant caller, including NanoSV v1.2.4 (Cretu Stancu, et al., 2017), SVIM v1.4.2 (Heller and Vingron, 2019), and CuteSV v1.0.13 (Jiang, et al., 2020). Low-quality SV variants are filtered by using the "--minqual_sv" parameter, achieved through using SVIM. VD applies a default minimum quality threshold of 15, based on a previous publication (Lesack, et al., 2022). Similar to its SNP/INDEL calling module, VD provides great flexibility by accepting VCF files from other SV callers as input for the combineVariants module. This allows for the creation of a consensus set from different VCF files than those created by VD, catering to specific user needs.

SURVIVOR v1.0.7 (Jeffares, et al., 2017) is used to generate intersections of all VCF files. The parameters for merging consensus structural variants are a maximum allowed distance of 1 kbp between breakpoints and calls supported by at least three variant callers (default) where there is agreement on both type and strand.

- 1. **Output**

Consensus VCF files from either tool are converted into additional comma-separated values (CSV) and tab-separated values (TSV) files. Additionally, a summary text file is generated for each tool, which provides information on the number of occurrences for each variant type found in the sample.

1. **Generation of simulation datasets**

Simulated datasets were generated by SURVIVOR v1.0.7 (Jeffares, et al., 2017) using the genome assembly of *Escherichia coli* (*E. coli*) str. K-12 substr. MG1655 ([NC_000913.3](https://www.ncbi.nlm.nih.gov/nuccore/NC_000913.3)), *Burkholderia mallei* (*B. mallei*) str. ATCC 23344 ([GCF_000011705.1](https://www.ncbi.nlm.nih.gov/datasets/genome/GCF_000011705.1/)), and *Lactobacillus acidophilus* (*L. acidophilus*) str. La-14 ([CP005926.2](https://www.ncbi.nlm.nih.gov/nuccore/CP005926.2/)) for the evaluation of VD’s performance. These bacterial species were chosen to represent a spectrum of genomic GC contents, providing a comprehensive assessment of VD’s performance across different bacterial genomes. *L. acidophilus*, with a low GC content of 34.5%, and *B. mallei*, with a high GC content of 68.5%, complement the medium GC content of *E. coli* (50.5%). The simulation process consisted of two rounds. In the first round, SNPs were introduced, while in the second round, SVs were introduced. This approach ensured that SNPs were not added to insertions introduced in the original genome. SNPs that were removed due to deletions were manually removed from the datasets to ensure accuracy. Overall, five independent datasets for each species were generated for evaluation purposes. Each dataset had an SNP mutation rate of 0.00017 variants per base pair. The SNP amounts in each bacteria were approximately 750, 1000, and 300 for *E. coli*, B. mallei, and *L. acidophilus*, respectively. Additionally, 100 SVs were introduced into each genome. The SVs were further categorized into distinct groups and sizes, including 85 insertions/deletions ranging from 1,000 to 30,000 base pairs, 3 duplications spanning 1,000 to 20,000 base pairs, 1 translocation (counts as 2 breakpoints) spanning 10,000 to 30,000 base pairs (in *B. mallei* only), and 10 inversions ranging from 500 to 10,000 base pairs. Numbers were based on variant rates observed in 17 *B. mallei* completed and chromosome-level genome assemblies available in the RefSeq database (2023-05-01), which contain both short-read and long-read raw data (Supplemental Table S1). Since the genome size of *L. acidophilus* was considerably smaller (1.99 Mbp) in comparison to the *E.coli* (4.64 Mbp) and *B. mallei* (5.84 Mbp) genomes, all SV sizes (in bp) were reduced by a factor of 3.

1. **Benchmarking using simulation datasets**

The simulated datasets were used to conduct multiple timing benchmarks to assess the performance of VD. These benchmarks included measuring the runtime against the number of threads (Figure S2), read coverage (Figure S3), and the number of variants (Figure S4). Additionally, benchmarking was carried out to evaluate VD's precision, recall, and F1 scores in order to determine the optimal number of variant callers required to generate the consensus file (Figure S5). These benchmarks also involved comparing VD`s performance against other tools. All the scripts used in the benchmarking process can be found in the “benchmarking” directory within VD’s GitHub repository (<https://github.com/OLF-Bioinformatics/VariantDetective>).

Additional benchmarking was conducted to assess the impact of mapping simulated reads to the original genome, where *in silico* mutations were introduced, and to a “divergent” reference genome. For this analysis, we use simulated data and truth sets from a prior study by Bush et al. (2020), which employed a similar workflow for evaluating other pipelines. We selected 29 *E. coli* genomes from their dataset to gauge the performance of VD in mapping reads to either the same or a divergent genome.

1. **Benchmarking using real datasets**

Eighteen datasets from a previous study (Bush, et al., 2020) were used to benchmark VD’s performance with SNP/INDEL from real sequencing data (<https://ora.ox.ac.uk/objects/uuid:8f902497-955e-4b84-9b85-693ee0e4433e>). This data was used to generate precision, recall, and F1 scores for VD when compared to other variant callers. PacBio sequencing reads from the Coriell NA12878 human sample (Genomes Project, et al., 2015) were used to investigate performances of SV prediction (<ftp://ftp.1000genomes.ebi.ac.uk/vol1/ftp/technical/working/20131209_na12878_pacbio/si/>). The high-confidence SV benchmarking results of this dataset have been reported previously (Parikh, et al., 2016). However, due to the incomplete nature of this SV benchmark set, only the recall performance could be estimated. All the scripts used in the benchmarking process, including those used to compile and evaluate the results, can be found in the “benchmarking” directory within VD’s GitHub repository (<https://github.com/OLF-Bioinformatics/VariantDetective> ).

**Supplementary Table**

**Supplementary Table S1 Genetic variant calling of *B. mallei* strains using VD**

| **Strain^a^** | **SRA_ILL** | **SRA_PACBIO_ONT** | **SNP/INDEL** | | | | | | | | **SV** | | | |  | | |
| --- | --- | --- | --- | --- | --- | --- | --- | --- | --- | --- | --- | --- | --- | --- | --- | --- | --- |
|  |  |  | **TOTAL** | **SNP** | **DEL** | **INS** | **MNP** | **COM** |  | **TOTAL** | **TRA** | **INV** | **DEL** | **INS** | | **DUP** |  |
| FMH 23344 | SRR1618492 | SRR1618494 | 27 | 15 | 6 | 4 | 1 | 1 |  | 1 | 0 | 1 | 0 | 0 | | 0 |  |
| 6 | SRR1618671 | SRR1618669 | 1774 | 1340 | 201 | 170 | 24 | 39 |  | 157 | 1 | 9 | 80 | 67 | | 0 |  |
| BMQ | SRR1618688 | SRR1618686 | 1082 | 815 | 162 | 85 | 1 | 19 |  | 117 | 0 | 6 | 60 | 51 | | 0 |  |
| 2000031063 | SRR1618499 | SRR1618500 | 732 | 534 | 106 | 72 | 7 | 13 |  | 65 | 0 | 9 | 51 | 3 | | 2 |  |
| 2002734299 | SRR1618349 | SRR1618350 | 1218 | 909 | 164 | 109 | 7 | 29 |  | 103 | 1 | 5 | 50 | 47 | | 0 |  |
| 11 | SRR1616952 | SRR1617359 | 1270 | 952 | 185 | 103 | 11 | 19 |  | 96 | 1 | 11 | 68 | 15 | | 1 |  |
| India86-567-2 | SRR2146904 | SRR2146906 | 893 | 662 | 138 | 74 | 5 | 14 |  | 116 | 0 | 17 | 69 | 29 | | 1 |  |
| 2002734306 | SRR2146899 | SRR2146901 | 1656 | 1287 | 168 | 149 | 22 | 30 |  | 119 | 1 | 16 | 90 | 11 | | 1 |  |
| KC_1092 | SRR2146902 | SRR2146903 | 644 | 526 | 71 | 36 | 1 | 10 |  | 58 | 0 | 6 | 49 | 3 | | 0 |  |
| 2002721276 | SRR2147667 | SRR2147669 | 649 | 476 | 86 | 70 | 2 | 15 |  | 65 | 0 | 1 | 35 | 29 | | 0 |  |
| FDAARGOS_585 | SRR8283094 | SRR8283092 | 1133 | 822 | 153 | 120 | 6 | 32 |  | 94 | 1 | 14 | 72 | 4 | | 3 |  |
| FDAARGOS_588 | SRR8072932 | SRR8072934 | 1199 | 862 | 169 | 130 | 9 | 29 |  | 144 | 0 | 7 | 80 | 56 | | 1 |  |
| FDAARGOS_589 | SRR8072935 | SRR8072936 | 867 | 610 | 139 | 91 | 5 | 22 |  | 80 | 0 | 10 | 65 | 5 | | 0 |  |
| FDAARGOS_590 | SRR8072938 | SRR8072939 | 30 | 15 | 7 | 5 | 1 | 2 |  | 1 | 0 | 0 | 0 | 0 | | 1 |  |
| 34 | ERR9616711 | ERR9616715 | 1277 | 903 | 183 | 155 | 7 | 29 |  | 161 | 0 | 11 | 74 | 76 | | 0 |  |
|  |  | **AVERAGE** | **963.4** | 715.2 | 129.2 | 91.5 | 7.3 | 20.2 |  | **91.8** | 0.3 | 8.2 | 56.2 | 26.4 | | 0.7 |  |

1. *B. mallei* completed and chromosome-level genome assemblies available in the RefSeq database(2023-05-01), which contain both short-read and long-read raw data. SNP: Single Nucleotide Polymorphism; SV: Structural Variant; MNP: Multiple Nucleotide Polymorphism; INS: Insertion; DEL: Deletion; COM: Complex (combination of SNP/MNP); TRA: Translocation; INV: Inversion; CON: Contraction; DUP: Duplication.

**Supplementary Table S2 Comparative performance analysis of VD and other callers using *B. mallei* simulated long reads.**

| Simulated  data | Caller | Total  SV | FN | | | | |  | FP | | | | |
| --- | --- | --- | --- | --- | --- | --- | --- | --- | --- | --- | --- | --- | --- |
|  |  |  | DEL | DUP | INV | TRA | INS |  | DEL | DUP | INV | TRA | INS |
| 1 | VD | 100 | 2 | 0 | 0 | 0 | 0 |  | 0 | 0 | 0 | 0 | 2 |
|  | NanoVar | 100 | 0 | 0 | 0 | 1 | 0 |  | 0 | 0 | 8 | 2 | 6 |
|  | NanoSV | 100 | 12 | 0 | 0 | 0 | 0 |  | 8 | 0 | 16 | 0 | 9 |
|  | CuteSV | 100 | 0 | 0 | 0 | 0 | 0 |  | 6 | 1 | 4 | 0 | 9 |
|  | SVIM | 100 | 4 | 1 | 2 | 0 | 16 |  | 0 | 0 | 0 | 0 | 5 |
| 2 | VD | 100 | 2 | 0 | 0 | 0 | 1 |  | 2 | 0 | 0 | 0 | 1 |
|  | NanoVar | 100 | 0 | 0 | 0 | 0 | 3 |  | 1 | 4 | 3 | 0 | 7 |
|  | NanoSV | 100 | 9 | 1 | 0 | 0 | 0 |  | 3 | 0 | 12 | 0 | 4 |
|  | CuteSV | 100 | 1 | 0 | 0 | 0 | 1 |  | 4 | 1 | 1 | 0 | 3 |
|  | SVIM | 100 | 2 | 1 | 2 | 0 | 27 |  | 2 | 0 | 0 | 0 | 2 |
| 3 | VD | 100 | 0 | 0 | 0 | 0 | 5 |  | 0 | 0 | 0 | 0 | 0 |
|  | NanoVar | 100 | 0 | 0 | 0 | 0 | 4 |  | 0 | 3 | 11 | 1 | 6 |
|  | NanoSV | 100 | 12 | 0 | 0 | 0 | 0 |  | 0 | 0 | 17 | 2 | 5 |
|  | CuteSV | 100 | 0 | 0 | 0 | 0 | 1 |  | 0 | 1 | 6 | 2 | 3 |
|  | SVIM | 100 | 1 | 1 | 2 | 0 | 15 |  | 0 | 0 | 0 | 0 | 1 |
| 4 | VD | 100 | 2 | 0 | 1 | 0 | 1 |  | 0 | 0 | 0 | 0 | 2 |
|  | NanoVar | 100 | 2 | 0 | 1 | 0 | 2 |  | 2 | 2 | 8 | 4 | 9 |
|  | NanoSV | 100 | 14 | 0 | 0 | 0 | 0 |  | 1 | 0 | 19 | 1 | 2 |
|  | CuteSV | 100 | 0 | 0 | 0 | 0 | 0 |  | 1 | 0 | 2 | 0 | 3 |
|  | SVIM | 100 | 2 | 0 | 2 | 0 | 15 |  | 0 | 0 | 0 | 0 | 2 |
| 5 | VD | 100 | 3 | 0 | 1 | 0 | 0 |  | 2 | 0 | 0 | 0 | 1 |
|  | NanoVar | 100 | 0 | 0 | 0 | 0 | 3 |  | 0 | 0 | 15 | 0 | 3 |
|  | NanoSV | 100 | 9 | 0 | 0 | 0 | 0 |  | 8 | 0 | 30 | 0 | 1 |
|  | CuteSV | 100 | 0 | 0 | 0 | 0 | 0 |  | 12 | 1 | 2 | 0 | 2 |
|  | SVIM | 100 | 3 | 1 | 1 | 0 | 17 |  | 9 | 0 | 0 | 0 | 1 |

SV: Structural Variant; DEL: Deletion; DUP: Duplication; INV: Inversion; TRA: Translocation; INS: Insertion.

**Supplementary Figures**


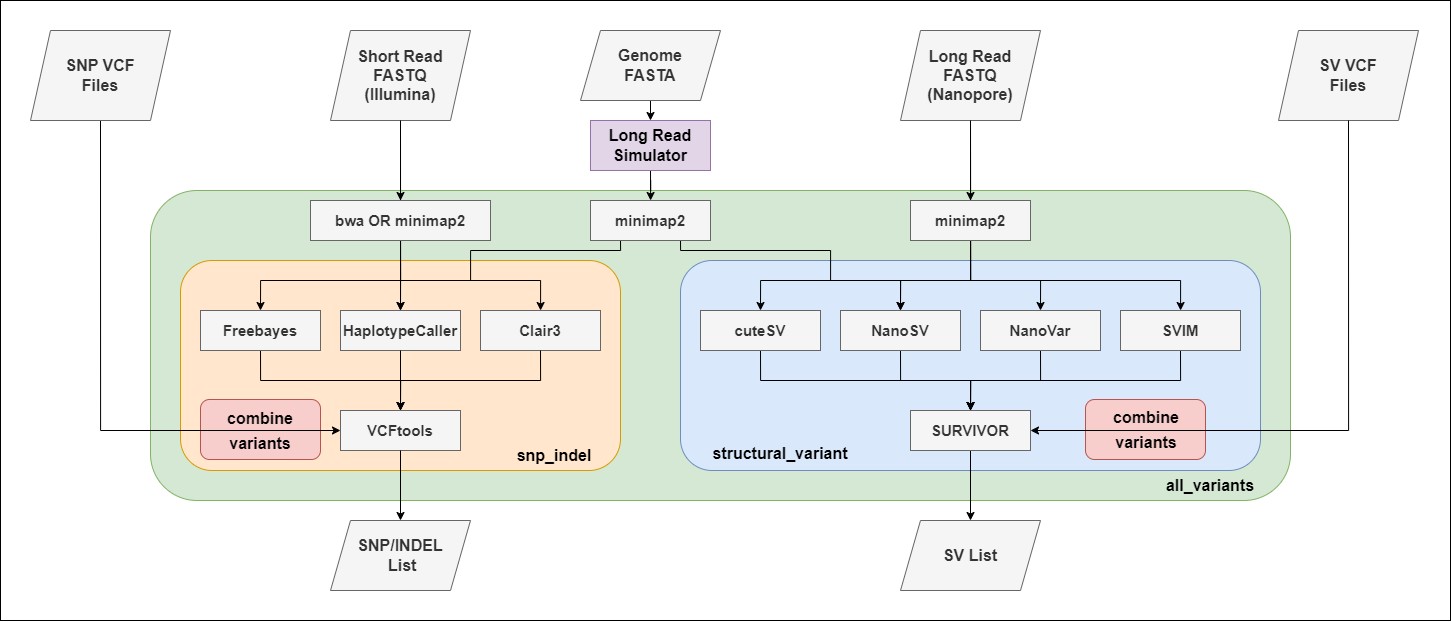


**Supplementary Figure S1. Overview of VariantDetective (VD) algorithm**.

The VD pipeline consists of two key modules including SNP/INDEL calling (yellow box) and SV calling (blue box), which can be executed independently or simultaneously. To initiate VD, the input requires a reference genome (FASTA) and query data in the form of short paired-end Illumina reads (FASTQ), long (Nanopore or PacBio) reads (FASTQ), or an assembled genome (FASTA). Once these files are supplied, data preparation involves generating all necessary supplementary files and, in the case of an assembled genome being provided as the query input, long reads will be simulated to allow further analyses. If both types of FASTQ reads or an assembled genome are provided as query input, all variants (as depicted in the green box) can be identified and analyzed. These variants are filtered, processed, and combined into consensus sets based off parameters specified when running VD. A fourth module in the VD toolkit (red boxes) allows to combine VCF files from different variants callers directly using a consensus-based approach.


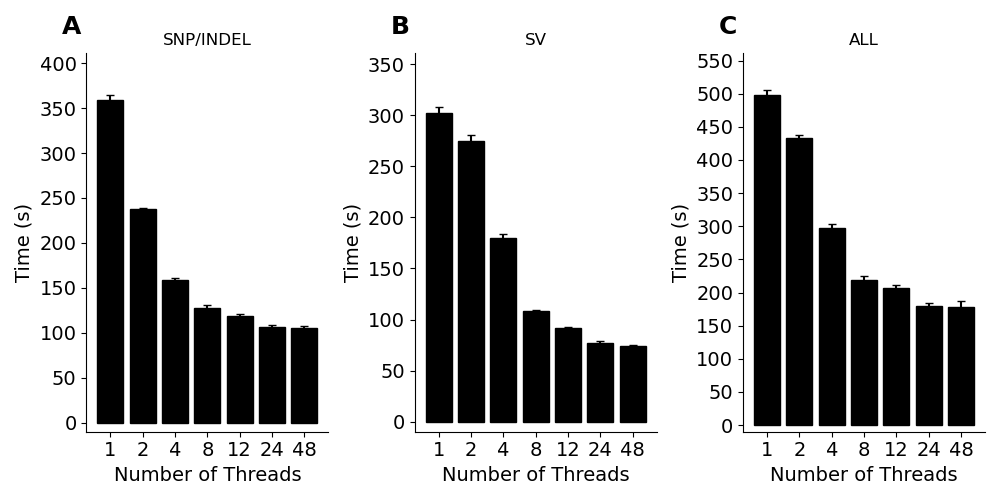


**Supplementary Figure S2. VD runtime using varying number of threads to predict genomic variants.**

(**A**) SNP/INDEL analysis where approximately 1000 SNPs were simulated in the genome. (**B**) SV analysis where 100 SVs were introduced in the genome. (**C**) The combined SNP/INDEL and SV analyses where genomes contained approximately 1000 SNPs and 100 SVs. For all analyses, five independent simulated datasets with 50X read coverage an Intel(R) Xeon(R) Silver 4214R CPU @ 2.40GHz were used to evaluate run time. The error bars represent the standard deviation across the datasets.

**
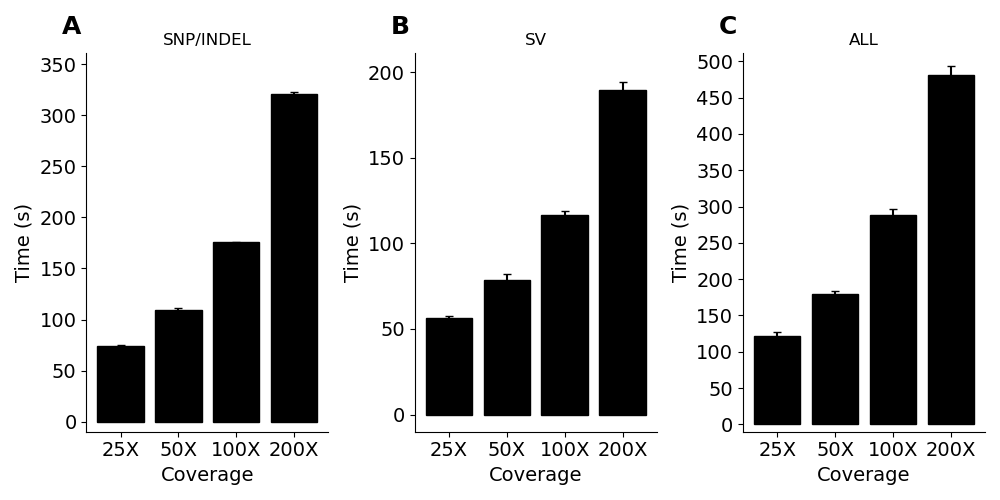
**

**Supplementary Figure S3. VD runtime using various sequencing coverages to predict genomic variants.**

(**A**) SNP/INDEL analysis where approximately 1000 SNPs were simulated in the genome. (**B**) SV analysis where 100 SVs were introduced in the genome. **(C)** the combined SNP/INDEL and SV analyses where genomes contained approximately 1000 SNPs and 100 SVs. For all analyses, five independent mutated datasets were simulated and variants were predicted using 24 threads with an Intel(R) Xeon(R) Silver 4214R CPU @ 2.40GHz to evaluate run time. The error bars represent the standard deviation across the datasets.

**
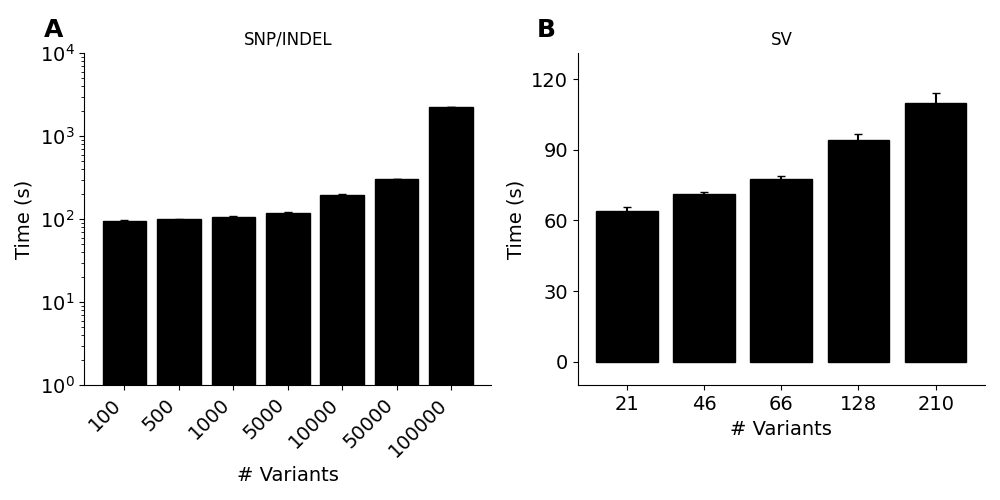
**

**Supplementary Figure S4. VD runtime using various number of genomic variants.**

(**A**) SNP/INDEL analysis where a range of SNP counts were simulated in the genome. (**B**) SV analysis where a range of SVs were introduced in the genome. For all analyses, five independent mutated datasets were simulated, each with a read coverage of 50X, and variant predictions were performed using 24 threads with an Intel(R) Xeon(R) Silver 4214R CPU @ 2.40GHz to assess run time. The error bars represent the standard deviation across the datasets.


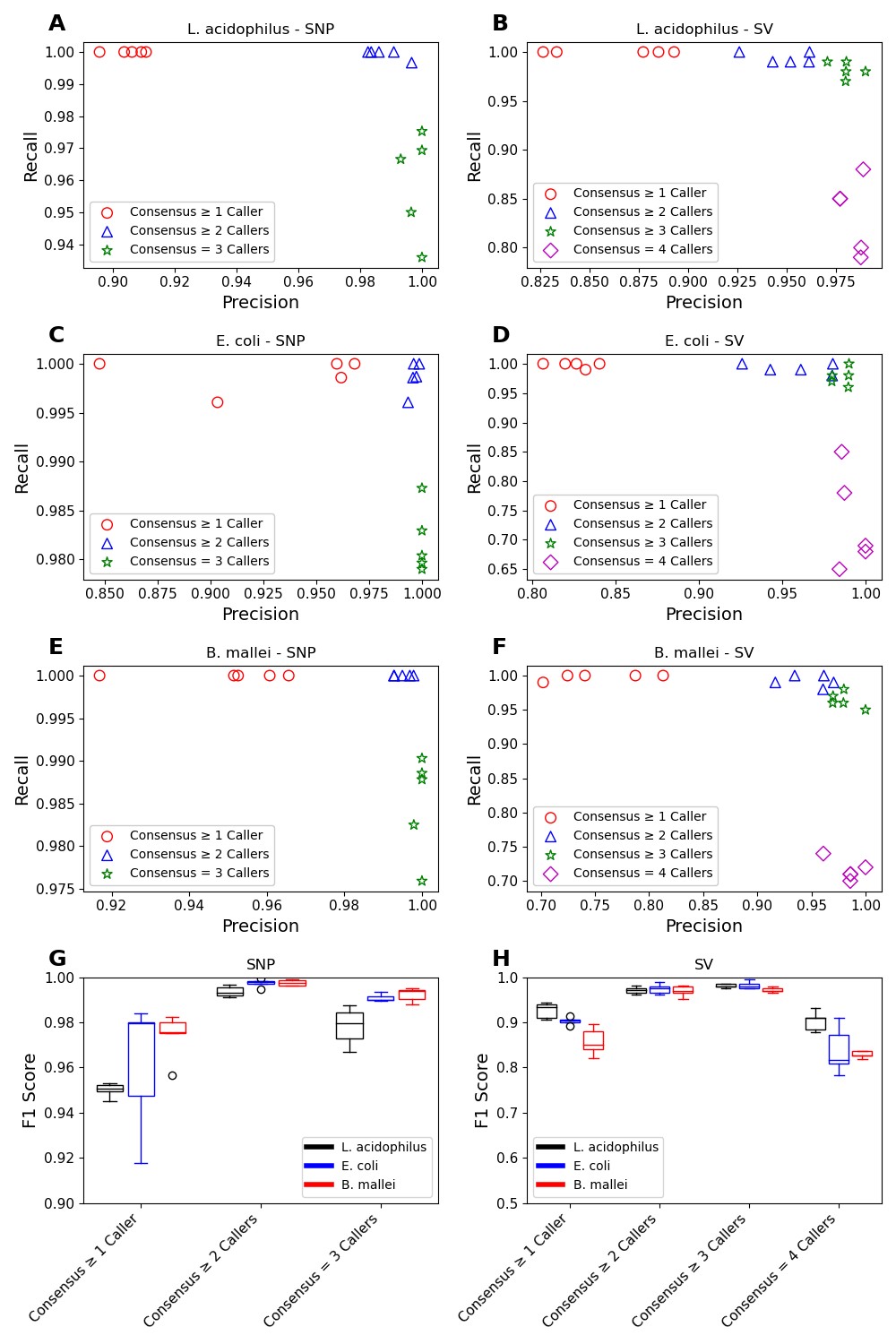


**Supplementary Figure S5. Performance of consensus calling results.**

**A and B.** VD precision and recall for SNP/INDEL and SV prediction for *L. acidophilus*. **C and D**. VD precision and recall for SNP/INDEL and SV prediction for *E. coli*. **E and F.** VD precision and recall for SNP/INDEL and SV prediction for *B. mallei*. **G and H**. VD performance for all three species for SNP/INDEL and SV prediction. Optimization of consensus variant sets were performed using five independent simulated datasets for each bacteria. Required support from different number of variant callers was assessed to determine default parameters to be used by the pipeline.

**Supplementary Figure S6. F1 scores of four SNP callers benchmarked against 29 *E. coli* SNP benchmarking datasets when the reference genome for alignments is (A) the same as the source of the reads and (B) a representative (divergent) genome for that species.**

The benchmarking datasets contained simulated sequencing data. Note that because the range of F1 scores were different between both cases, the vertical axes on each panel have different scales. VD: VariantDetective; FB: Freebayes; HC: HaplotypeCaller; CL: Clair3.

**Supplementary Figure S7. F1 scores of four SNP callers benchmarked against eighteen bacterial SNP benchmarking datasets using short-read sequencing data (FASTQ files, A) and assembled genomes (FASTA files, B).**

The benchmarking datasets contained real sequencing data from various bacterial genomes. VD: VariantDetective; FB: Freebayes; HC: HaplotypeCaller; CL: Clair3.

**Supplementary Figure S8. Performance of VD using aligner minimap2 and BWA against eighteen bacterial SNP benchmarking datasets**.

Metrics used for benchmarking: **(A)** Recall, (**B**) Precision, and (**C**) F1 score.

**Supplementary Figure S9. Comparison of VD’s F1 scores across different input types.**

The 18 bacterial SNP benchmarking datasets were used. Input types were short-read sequencing data (red) and assembled genomes (blue).

**References**

Barbitoff, Y.A.*, et al.* Systematic benchmark of state-of-the-art variant calling pipelines identifies major factors affecting accuracy of coding sequence variant discovery. *BMC Genomics* 2022;23(1):155.

Bolognini, D. and Magi, A. Evaluation of Germline Structural Variant Calling Methods for Nanopore Sequencing Data. *Front Genet* 2021;12:761791.

Bush, S.J.*, et al.* Genomic diversity affects the accuracy of bacterial single-nucleotide polymorphism-calling pipelines. *Gigascience* 2020;9(2).

Cretu Stancu, M.*, et al.* Mapping and phasing of structural variation in patient genomes using nanopore sequencing. *Nat Commun* 2017;8(1):1326.

Garrison, E. and Marth, G.J.a.p.a. Haplotype-based variant detection from short-read sequencing. 2012.

Genomes Project, C.*, et al.* A global reference for human genetic variation. *Nature* 2015;526(7571):68-74.

Heller, D. and Vingron, M. SVIM: structural variant identification using mapped long reads. *Bioinformatics* 2019;35(17):2907-2915.

Jeffares, D.C.*, et al.* Transient structural variations have strong effects on quantitative traits and reproductive isolation in fission yeast. *Nat Commun* 2017;8:14061.

Jiang, T.*, et al.* Long-read sequencing settings for efficient structural variation detection based on comprehensive evaluation. *BMC Bioinformatics* 2021;22(1):552.

Jiang, T.*, et al.* Long-read-based human genomic structural variation detection with cuteSV. *Genome Biol* 2020;21(1):189.

Lesack, K.*, et al.* Different structural variant prediction tools yield considerably different results in Caenorhabditis elegans. *PLoS One* 2022;17(12):e0278424.

Li, H. Minimap and miniasm: fast mapping and de novo assembly for noisy long sequences. *Bioinformatics* 2016;32(14):2103-2110.

Li, H. and Durbin, R. Fast and accurate short read alignment with Burrows-Wheeler transform. *Bioinformatics* 2009;25(14):1754-1760.

Li, H.*, et al.* The Sequence Alignment/Map format and SAMtools. *Bioinformatics* 2009;25(16):2078-2079.

Liu, Y.H.*, et al.* Methods for structural variant detection with long-read sequencing data. In.: Research Square; 2022.

McKenna, A.*, et al.* The Genome Analysis Toolkit: a MapReduce framework for analyzing next-generation DNA sequencing data. 2010;20(9):1297-1303.

Molina-Mora, J.A. and Solano-Vargas, M. Set-theory based benchmarking of three different variant callers for targeted sequencing. *BMC Bioinformatics* 2021;22(1):20.

Parikh, H.*, et al.* svclassify: a method to establish benchmark structural variant calls. *BMC Genomics* 2016;17:64.

Romagnoli, S., Bartalucci, N. and Vannucchi, A.M. Resolving complex structural variants via nanopore sequencing. *Front Genet* 2023;14:1213917.

Smolka, M.*, et al.* Detection of mosaic and population-level structural variants with Sniffles2. *Nat Biotechnol* 2024.

Tham, C.Y.*, et al.* NanoVar: accurate characterization of patients' genomic structural variants using low-depth nanopore sequencing. *Genome Biol* 2020;21(1):56.

Wang, Y.*, et al.* Nanopore sequencing technology, bioinformatics and applications. *Nat Biotechnol* 2021;39(11):1348-1365.

Yang, L. A Practical Guide for Structural Variation Detection in the Human Genome. *Curr Protoc Hum Genet* 2020;107(1):e103.

Zheng, Z.*, et al.* Symphonizing pileup and full-alignment for deep learning-based long-read variant calling. *Nature Computational Science* 2022;2(12):797-803.
